# Enhanced Anti-Biofouling Properties of BWRO Membranes via the Deposition of Poly (Catechol/Polyamine) and Ag Nanoparticles

**DOI:** 10.3390/membranes13050530

**Published:** 2023-05-19

**Authors:** Lixin Xie, Yaqian Liu, Shichang Xu, Wen Zhang

**Affiliations:** School of Chemical Engineering and Technology, Tianjin Key Laboratory of Membrane Science and Desalination Technology, State Key Laboratory of Chemical Engineering Tianjin University, Tianjin 300350, China; xie_lixin@tju.edu.cn (L.X.); liuyaqian8190@tju.edu.cn (Y.L.)

**Keywords:** RO membrane, anti-fouling, co-deposition, poly (catechol/polyamine), silver nanoparticles

## Abstract

The surface modification of reverse osmosis (RO) membranes to improve their anti-biofouling properties is gaining increased attention. Here, we modified the polyamide brackish water reverse osmosis (BWRO) membrane via the biomimetic co-deposition of catechol (CA)/tetraethylenepentamine (TEPA) and in situ growth of Ag nanoparticles. Ag ions were reduced into Ag nanoparticles (AgNPs) without extraneous reducing agents. The hydrophilic property of the membrane was improved, and the zeta potential was also increased after the deposition of poly (catechol/polyamine) and AgNPs. Compared with the original RO membrane, the optimized PCPA3-Ag10 membrane showed a slight reduction in water flux, and the salt rejection declined, but enhanced anti-adhesion and anti-bacterial activities were observed. The *FDR_t_* of the PCPA3-Ag10 membranes during the filtration of BSA, SA and DTAB solution were 5.63 ± 0.09%, 18.34 ± 0.33% and 34.12 ± 0.15%, respectively, much better than those of the original membrane. Moreover, the PCPA3-Ag10 membrane exhibited a 100% reduction in the number of viable bacteria (B. subtilis and E. coli) inoculated on the membrane. The stability of the AgNPs was also high enough, and these results verify the effectiveness of poly (catechol/polyamine) and the AgNP-based modification strategy for the control of fouling.

## 1. Introduction

Reverse osmosis (RO) membrane separation technology is considered a very promising technology for desalination and wastewater treatment. However, a significant obstacle to the high performance of RO technology is membrane biofouling, which impacts the water flux and the lifetime of membrane filters and modules [1,2,3,4,5]. Recently, several methods have been developed to monitor biofouling in RO systems, such as assimilable organic carbon (AOC), the membrane biofilm formation rate (mBFR) and bacterial growth potential (BGP) [6,7]. Many pretreatment processes have also been employed to decrease the fouling of RO membranes, such as the chlorination of feed water, ultrafiltration and microfiltration [8,9,10]. However, due to the self-replicating properties of bacteria, the above methods cannot completely reduce biofouling. Another strategy to address this problem is developing membranes with anti-bacterial properties on the membrane surface [11,12]. An anti-bacterial membrane can continuously inactivate bacteria and suppress biofilm formation, lowering the biofouling potential inside membrane modules during filtration.

Silver nanoparticles (AgNPs), due to the highly effective bactericides, have recently been widely used as anti-bacterial additives in the preparation of anti-biofouling RO membranes [13,14]. An early study on the preparation of AgNP-loaded RO membranes embedded AgNPs into the membrane matrix via the interfacial polymerization process [15,16,17,18]. However, this method usually involves complicated procedures and causes damage to separation layer structures. In addition, only a small percentage of AgNPs are exposed on the surface, and thus, the utilization of these AgNPs is low [17,19]. Therefore, the strategy of directly loading AgNPs on membrane surfaces has been developed to enhance the accessibility of these nanoparticles. In this strategy, there are usually appropriate sites on the membrane surfaces to bind and immobilize AgNPs. For example, Zhang et al. [20] immobilized AgNPs on membrane surfaces via polyacrylic acid sites. It was also reported that anti-bacterial RO membranes with cysteamine (NH2-(CH2)2-SH) sites can interact with AgNPs [21], AgNP-decorated silica particles [22] and AgNP -oxide nanosheets [23] through the covalent Ag-S bond. However, this strategy is restricted to limited grafting sites, which causes the low loading density of AgNPs.

Recently, polydopamine, a hydrophilic polymer with abundant hydroxyl and amine groups, has been widely used in the surface modification of RO membranes to reduce the adhesion properties of contaminants. In addition, polydopamine can also be employed as a reducing agent so that AgNPs can be easily incorporated onto polydopamine-coated membranes via the in situ reduction method [24]. However, the high cost of dopamine limits its extensive application in membrane surface modification [25]. Besides polydopamine, other polymer coatings, such as tannic acid-Fe^3+^-polyethyleneimine (TA-Fe-PEI), have also been used for the surface modification of RO membranes and the in situ generation of AgNPs to enhance the antibacterial properties [26,27]. These works are encouraging and help us to develop new substitutions for dopamine coatings to produce AgNPs on RO membranes. It is confirmed that catechol and polyamine play important roles in dopamine polymerization to form strong adhesion [28], and a low-cost poly(catechol-polyamine) (PCPA) coating has also been developed on the surface of polypropylene membranes without significant agglomeration. Just recently, PCPA modification with the co-deposition of AgNPs has been reported on the surface of various matrixes, including PVDF membranes [29], TiO_2_ [30] and aluminum beads [31]. However, to the best of our knowledge, there are few studies about the modification of RO membranes via the combination of PCPA coatings and AgNPs.

Therefore, based on the anti-adhesive property of PCPA and anti-bacterial property of AgNPs, we prepared a PCPA coating and studied AgNPs’ in situ growth on the surface of a commercial RO membrane. We demonstrated the successful loading of AgNPs on the surface and systematically tested the membrane properties. Then, we verified the anti-adhesion and anti-bacterial performance of the membrane. Finally, the stability of the AgNPs was tested using the static soaking method.

## 2. Experimental Section

### 2.1. Materials

Commercial brackish water reverse osmosis (BWRO) membrane (ESPA1) was purchased from Nitto Denko Hydranautics. Sodium bisulfite (NaHSO_3_) and nitric acid (HNO_3_) were obtained from Chemart Chemical Technology in Tianjin. Catechol (CA) (Aladdin, Shanghai, China), tris(hydroxymethyl)-aminomethane (Tris) (Aladdin, Shanghai, China) and Tetraethylenepentamine (TEPA) (Xianding biotechnology, Shanghai, China) were used for the crosslinking of the PCPA coating on the membrane surface. Sodium chloride (NaCl) (Chemart Chemical, Tianjin, China), bovine serum albumin (BSA) (Aladdin Bio-Chem Technology, Shanghai, China), sodium alginate (SA) (Meryer Chemical, Shanghai, China) and dodecyl trimethyl ammonium bromide (DTAB) (Meryer Chemical Technology, Shanghai, China) were used in the anti-adhesion test. Polyvinylpyrrolidone (PVP, K16-18) was purchased from Macklin Biochemical Co. Ltd. (Shanghai, China). Yeast extract, peptone, agar and lysozyme were purchased from Aoboxing Biotech Co. Ltd. (Beijing, China). Silver nitrate (AgNO_3_) (Guangfu Chemical Technology, Tianjin, China) was used for the formation of AgNPs. Bacillus subtilis (*B. subtilis*) (TransGen, Beijing, China) and Escherichia coli (*E. coli*) (TransGen, Beijing, China) were used in the anti-bacterial experiment. Deionized (DI) water (Distilled Water Department of Yongqingyuan, Tianjin, China) was used in all the experiments.

### 2.2. PCPA Coating and In Situ Formation of AgNPs

**Figure 1:**
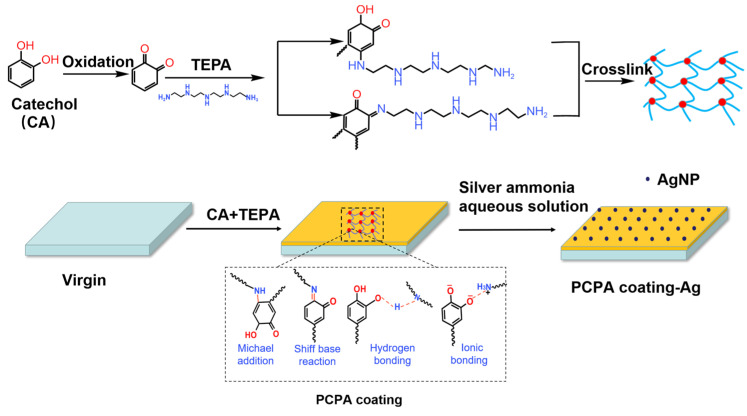
Schematic of the in situ formation of AgNPs on RO surface.

The preparation of the membranes consisted of two steps: the PCPA coating deposition and the in situ formation of AgNPs. The procedures used to prepare AgNP-loaded RO membranes are shown in Figure 1. The commercial membrane pieces were preserved in NaHSO_3_ solution (0.5 wt%). Before use, they were immersed in pure water for 1 day. The membrane pieces were fixed between the membrane frames (11 cm × 19 cm) with the active layer exposed to the modified solution. The modified solution was prepared by adding CA (2n mM, n = 2, 3, 4, 5, 6 and 7) and TEPA (n mM) into 40 mL of Tris-buffer (pH 8.5, 50 mM), and it was covered on the active layer of the RO membranes. The membrane frames was shaken on a rotating shaker for 15 min. The PCPA-coated membrane is denoted as *PCPAn*.

The PCPA3 membrane was washed with deionized water and then immersed in a mixture of solution containing of 15 mL of silver ammonia (*m* g·L^−1^, *m* = 5, 7.5, 10, 12.5 and 15) and 15 mL of PVP (1.5 g·L^−1^) for 15 min to obtain the membranes modified with AgNPs. The resulting membranes were referred to as *PCPA3-Agm*, where *m* is the concentration of silver ammonia solution. PVP was used as a dispersant and stabilizer to reduce the agglomeration of AgNPs. The above process was carried out on a shaker 85 times per minute. Finally, the *PCPA3-Agm* membranes were rinsed and kept in deionized water at 4 °C before use.

### 2.3. Characterization

X-ray photoelectron spectroscopy was used to characterize the chemical composition of the membrane surface (XPS, K-Alpha+, ESCALAB-250Xi, ThermoFisher Scientific, Loughborough, UK) with energy-dispersive X-ray spectroscopy (EDS, NanoSEM430, FEI, USA). The total number of AgNPs on the membranes was determined using Inductively Coupled Plasma Mass Spectrometry (ICP-MS, Agilent7700, Agilent, Santa Clara, CA, USA). Before the test, the membrane sample (3 cm × 3 cm) was digested using HNO_3_ solution. Then, the resulting solution was kept at 90 °C for 2 days to make all the AgNPs into Ag ions in the solution.

A field emission scanning electron microscope was used to characterize the surface morphology of the membranes (SEM S4800, Hitachi, Tokyo, Japan). Atomic force microscopy was employed to characterize the surface roughness (AFM, Dimension Icon, Bruker Co., Bremen, Germany). Before these tests, the samples were dried under vacuum at 60 °C for 1 day. The water contact angle was tested using an optical contact angle goniometer (OCA15EC, DataPhysics, Filderstadt, Germany). The water contact angle was tested at 10 different locations on one sample, and the average value was used. The Zeta potential of the surface was tested using a solid surface Zeta potential tester (SurPASS, Anton Paar GmbH, GmbH, Graz, Austria) using 1 mM KCl background solution at 25 °C.

### 2.4. Water Flux and Salt Rejection 

A cross-flow device was employed to assess the water flux and salt rejection of all the membranes. The area (A) of the membranes was 31.16 cm^2^. The permselectivity of the membrane was tested at 25 °C and 1.55 ± 0.1 MPa. The feed was 2000 mg·L^−1^ NaCl solution. The cross-flow rate of the feed was 1.5 L·min^−1^ [32]. The water flux (*J*, L·m^−2^·h^−1^) of the RO membrane was calculated using Equation (1):

(1) $$J=\frac{V}{A\cdot T}$$

in which *V* is the volume of permeated water (L), and *T* is the recorded measurement time (h). The salt rejection rates (*R*) of membranes were determined using Equation (2):

(2) $$R=\left(1-\frac{C_{p}}{C_{f}}\right)\times 100\%,$$

in which *C_p_* and *C_f_* are the salt concentrations of the permeated and feed solutions, respectively. The salt concentrations were tested using a conductivity instrument (DDSJ-308A).

### 2.5. Anti-Adhesion Property Evaluation

Three model pollutants, BSA (150 mg·L^−1^), SA (100 mg·L^−1^) and DTAB (50 mg·L^−1^), were employed to determine the anti-adhesion performance of the PCPA3-Ag10 membrane.

First, the RO membranes were compacted in a solution of 2000 mg·L^−1^ NaCl for 60 min at 1.55 ± 0.1 MPa to obtain a stable flow. Then, the initial pure water flux (*J*_0_) was obtained at the cross-flow rate of 1.5 L·min^−1^ for 0.5 h. After the filtration of the NaCl solution for 30 min, the model contaminants were admitted to feed solution for the anti-adhesion tests. The anti-adhesion tests lasted 4.5 h, and the water flux (*J_t_*) was recorded every 3 min. At the end of the filtration process, the virgin and PCPA3-Ag10 membranes were rinsed with DI water for 1 h with the cross-flow rate of 2 L·min^−1^ at 0.7 MPa. All membranes were tested in two filtration-rinse cycles [33].

The anti-adhesion performances of the membrane were described via the flux recovery rate after cleaning *(FRR_c_*, c = 1 or 2) and the flux decline rate (*FDR_t_*). The calculation Formulas (3) and (4) are offered as follows:

(3) $$FRR_{c}\;=\;\frac{J_{t,c}}{J_{0}}\times 100\%\;\left(c\;=\;1,2\right)$$

(4) $${FDR}_{t}\;=\;(1-\frac{J_{f}}{J_{0}})\times 100\%,$$

in which *J_t,c_* is the water flux of the RO membranes after first (*c* = 1) or second (*c* = 2) rinse, and *J_f_* is the water flux after the second filtration test.

### 2.6. Anti-Bacterial and Stability Property of Membranes

The anti-bacterial performance was assessed via the vitality of bacteria after touching the membrane surface. The freeze-dried *E. coli* and *B. subtilis* cultures were refreshed in an LB via 12 h incubation in a 37 °C shaking incubator. Then, 100 μL of the refreshed culture was diluted 10^3^ times with 10 mL of sterilized saline (0.9 wt%) to make a bacterial suspension. Membrane samples (2.5 cm × 7.6 cm) were sterilized under UV irradiation for 1 h before the test. In a Petri dish, membrane samples were placed on glass slides with the upward active layer. The 100 µL of *E. coli* suspension or *B. subtilis* suspension was dispersed on the surface of the membranes. The cell concentration of *E. coli* and *B. subtilis* subtilis suspension was approximately 1.0 × 10^6^ cfu·mL^−1^. Then, a cover glass slide was placed over the membrane surface to ensure full contact with the bacterial suspension. The Petri dish was kept in the incubator with the temperature of 37 °C for 1.5 h. The RO membrane samples and slides were fully rinsed with 20 mL of saline, and the wash solution was collected. Then, 100 µL of the wash solution was inoculated on the LB agar medium, and the number of bacterial colonies was marshalled after incubation in the incubator for 12 h. The mortality rate (*E*) was calculated using the following equation:

(5) $$E\;=\;\frac{\left(B-A\right)}{B}\times 100\%,$$

in which *A* and *B* is the number of viable bacteria grown on LB agar plates using the microbial suspension contacted with and without the membrane, respectively.

The stability of immobilized AgNPs on membranes was investigated via static leaching experiments according to the previous report [27]. The membrane samples (3 cm × 3 cm) were immersed in 5% NaHCO_3_ solution (pH 8.2). The Ag ion concentration in the solutions was determined using ICP-MS.

## 3. Results and Discussion

### 3.1. Membrane Characterizations

**Figure 2:**
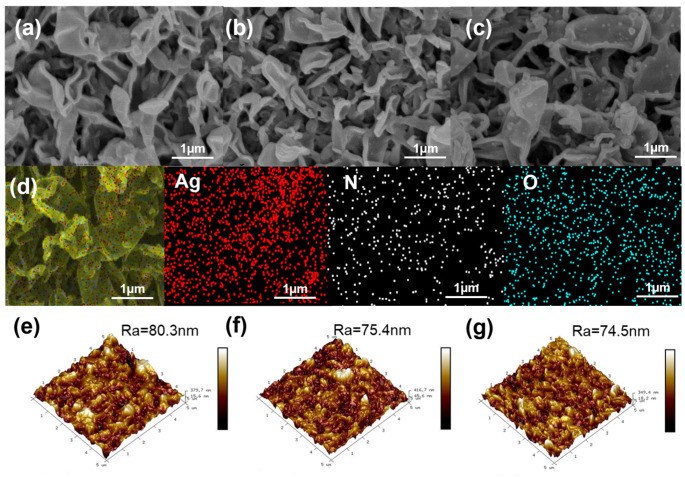
Surface SEM images of (**a**) virgin, (**b**) PCPA3 and (**c**) PCPA3-Ag10 membrane; (**d**) EDS mapping characterization of PCPA3-Ag10 membrane; AFM images of (**e**) virgin, (**f**) PCPA3 and (**g**) PCPA3-Ag10 membrane.

Figure 2a–c show the SEM micrographs of the virgin, PCPA3 and PCPA3-Ag10 membranes. All membranes had a distinct “ridge and valley” rough structure. The surface morphology of the PCPA3 membrane showed no significant change compared to the virgin one. However, after the immersion of PCPA3 in the silver ammonia solution, fine particles appeared on the membrane surface of PCPA3-Ag10.

We further investigated the loading amount of silver on the PCPA3-Ag10 membrane. The EDS elemental map images in Figure 2d show a clear signal of silver, nitrogen and oxygen elements, and the EDS elemental compositions show the atomic percent of Ag was 1.42% in Table 1.

According to the ICP-MS results, the total amount of AgNPs on the PCPA3-Ag10 membrane was 28.21 μg·cm^−2^. We also measured the total amount of AgNPs on the PCPA3-Agm membranes, and the results show that the total amount of AgNPs on the *PCPA3-Agm* membranes increased with the Ag concentration in silver ammonia solution (SI, Figure S1). The uniform PCPA interlayer could provide binding sites for the reduction of Ag ions, and the presence of catechol can reduce Ag ions into zero-valent AgNPs [24].

From the AFM images in Figure 2e–g, the average roughness (*Ra*) values of the virgin, PCPA 3 and PCPA3-Ag10 membranes were 80.3, 75.4 and 74.5 nm, respectively. Compared with the virgin membrane, the roughness of PCPA3 membranes was slightly reduced, and parts of the valley structures were filled. This is because the size of AgNPs is very small, and AgNPs have little contribution to the roughness of membranes. Hence, the PCPA3 and PCPA3-Ag10 membranes had similar roughness. Overall, the surface morphology of the RO membranes did not change much after the PCPA and AgNP modification.

**Figure 3:**
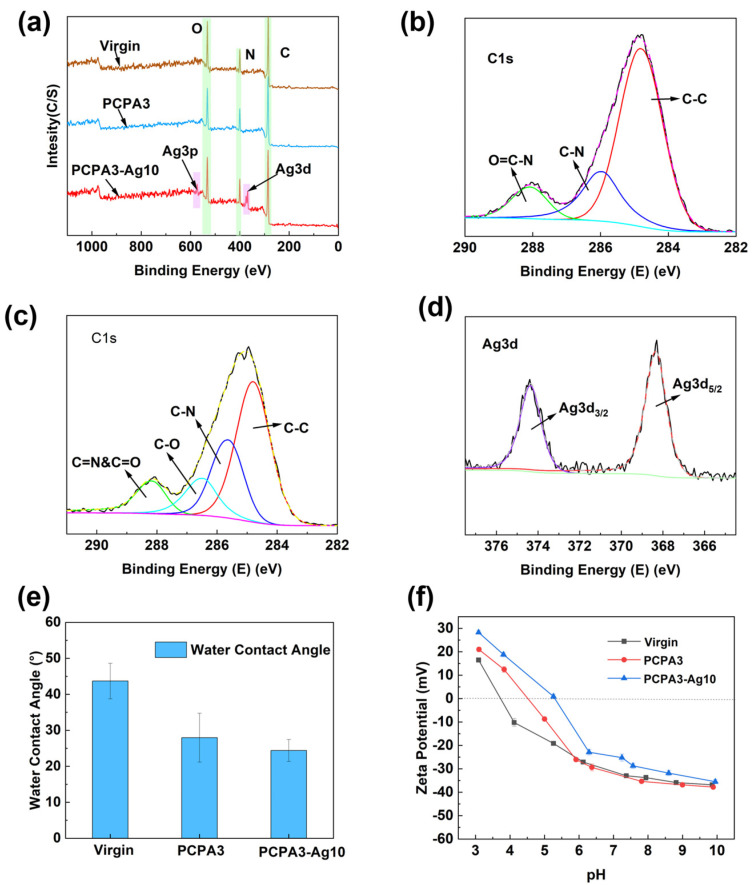
(**a**) XPS survey scans of membranes; C1s fine XPS spectra of the (**b**) virgin and (**c**) PCPA3 membrane; (**d**) Ag 3d spectra of the PCPA3-Ag10 membrane; (**e**) water contact angles of RO membranes and (**f**) zeta potentials of virgin, PCPA3 and PCPA3-Ag10 membranes.

The elemental compositions of membrane surfaces before and after the PCPA modification were captured using XPS. Figure 3a shows the full spectra of elements on the virgin, PCPA3 and PCPA3-Ag10 membrane surfaces. Table 2 shows the elemental composition and proportion of membranes via XPS, and the PCPA3 membrane had increased N/O content compared to the virgin membrane. This was attributed to the higher N content in the PCPA coating. The peak fits of C1s fine spectra from the virgin and PCPA3 membranes are shown in Figure 3b,c. Compared to the virgin membrane, the PCPA3 membrane showed a new peak at 286.6 eV due to the C-O bond, which was derived from the phenol structure in CA crosslinked on the membrane surface. In Figure 3d, the spectrum of the PCPA3-Ag10 membrane showed peaks for Ag 3d 3/2 and Ag 3d 5/2 with binding energies of 374.3 eV and 368.2 eV, which belonged to the peak of Ag^0^ [34]. 

The water contact angle was tested to evaluate the hydrophilicity of the membrane surface in Figure 3e. The water contact angle of the virgin membrane was 43.7 ± 4.9°. The PCPA3 membrane had smaller water contact angles than the virgin one due to the large numbers of hydrophilic amino and phenolic groups in the PCPA3 coating. The water contact angle of the PCPA3-Ag10 membrane was 24.7 ± 4.0°, which was attributed to the addition of AgNPs with high hydrophilicity. In addition, we also found that the water contact angle decreased continuously with the increase in Ag contents (SI, Figure S2).

The surface charge was demonstrated by measuring the streaming potential of the membrane surface in the range of pH 3–10. Figure 3f shows the amphoteric properties on the existing RO membrane surface. The Zeta potentials of the virgin membrane were 16.5 ± 0.9 mV at pH 3 and −36.8 ± 0.2 mV at pH 10. The zeta potentials of PCPA3 were 21.0 ± 0.9 mV at pH 3 and −37.8 ± 0.6 mV at pH 10. Additionally, the zeta potentials of PCPA3-Ag10 were 28.3 ± 0.8 mV at pH 3 and −35.5 ± 0.9 mV at pH 10. Compared to the virgin membrane, the Zeta potential of both modified membranes increased, with a rightward shift in the equipotential point. The PCPA coating contained a large amount of amine, which was protonated to increase the positive charge density under acidic conditions. Meanwhile, the partial dissolution of AgNP particles loaded on the membrane surface under acidic conditions may also have led to an enhanced positive charge on the surface of the membrane [14].

### 3.2. Membrane Permselectivity

**Figure 4:**
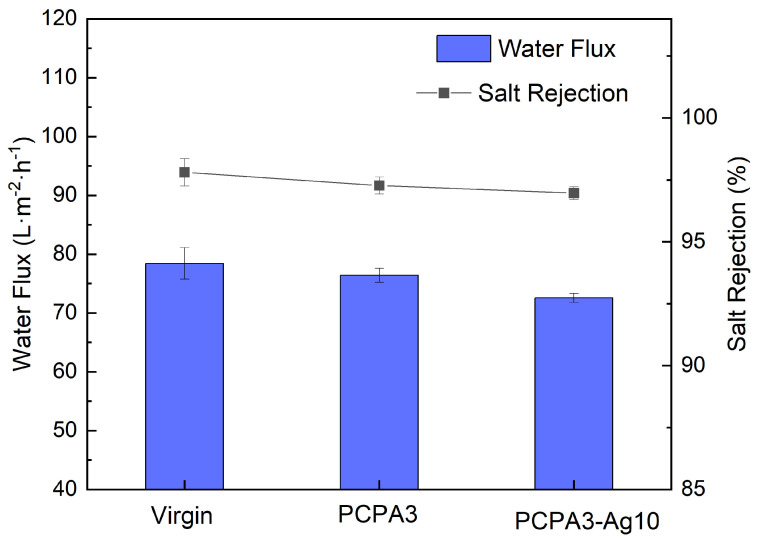
The water flux and salt rejection of the virgin, PCPA3 and PCPA3−Ag10 membranes.

To evaluate the permselectivity of the membranes, we investigated the water flux and salt rejection of the virgin, PCPA3 and PCPA3-Ag10 membranes. The water flux of the virgin membrane was 78.4 ± 2.7 L·m^−2^·h^−1^, as shown in Figure 4. The water flux of the PCPA3 and PCPA3-Ag10 membranes showed a certain decline in comparison to the virgin membrane. There were abundant polar groups (such as amino and hydroxyl groups) in the PCPA coating, which could have accelerated the penetration ability of water across the membrane. However, the PCPA coating could have also improved the mass transfer resistance of water across the membrane [29,35]. The water flux of the PCPA3-Ag10 membrane declined, because AgNPs could partly block water channels on the membrane surface [24]. The PCPA3 and PCPA3-Ag10 membranes showed a slight decrease in salt rejection in comparison to the virgin membrane. On the basis of the solution-diffusion model [36], whether the rejection of modification membrane was improved or decreased depended on the water/salt permeability ratio of modification membranes in comparison to the water/salt permeability ratio of the virgin one. The PCPA coating and the loading of AgNPs reduced the water permeability, leading to a low water/salt permeability ratio after modification [37,38].

### 3.3. Membrane Anti-Adhesion Properties

**Figure 5:**
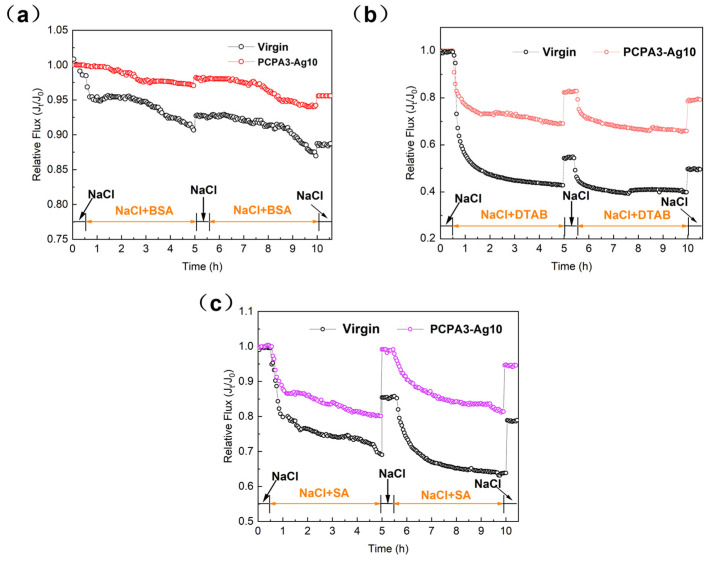
Relative flux of water across membranes tested with (**a**) 150 mg·L^−1^ BSA, (**b**) 50 mg·L^−1^ DTAB and (**c**) 100 mg·L^−1^ SA in 2000 mg·L^−1^ NaCl solution.

Three model pollutants, BSA (representing proteins in the water treatment process), SA (simulating polysaccharides) and DTAB (characterizing fouling surfactants in wastewater), were selected to study the anti-adhesion property of the PCPA3-Ag10 membrane. The relative flux *J_t_*/*J*_0_ was used to evaluate the change in flux for the membrane fouling and recovery. In Figure 5, it is shown that the relative flux of the membranes decreased with time in each cycle.

After washing, the water flux of all membranes recovered to some extent. The FRR_1_ values were always higher than the FRR_2_ values in all cycles, which could be attributed to the irreversible adsorption with pollutants. Compared to the virgin one, the relative flux of PCPA3-Ag10 membrane showed a lower loss of water flux. The higher *FRR_c_* (c = 1 and 2) and lower *FDR_t_* of the PCPA3-Ag10 membrane is shown in Table 3, indicating that this modification method can efficiently reduce the adsorption of pollutants.

The decreased degrees of water flux were different in the three contaminated environments. The relative flux in the BSA-contaminated environment had the lowest decline, while the relative flux in the DTAB-contaminated environment had the highest decline. DTAB, as a quaternary ammonium salt, has a positive charge in aqueous solutions. The membranes with negative charges had a stronger adsorption effect on DTAB than BSA and SA. This is the reason for the highest loss of water flux in the DTAB-contaminated environment.

### 3.4. Membrane Anti-Bacterial Property

**Figure 6:**
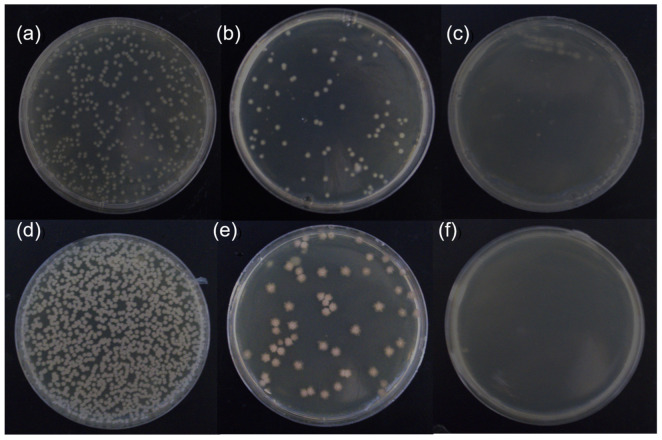
Photographs of LB agar of *E. coli* and *B. subtilis* colonies in contact with (**a**,**d**) virgin, (**b**,**e**) PCPA3 and (**c**,**f**) PCPA3-Ag10 membrane for 1.5 h. (inoculum level ca. 6.5 × 10^7^ cfu·m^−2^).

The anti-bacterial performance of the membranes was studied by growing two bacteria which were in contact with the membranes for 1.5 h. Figure 6 reveals the bacterial colonies grown on LB plates. The mortalities of the bacteria were obtained by counting the number of colonies, as shown in Table 4.

The mortalities of *B. subtilis* and *E. coli* which were in contact with the virgin membrane were 12.5 ± 1.7% and 15.5 ± 0.9%, respectively. Additionally, the mortality rates of *B. subtilis* and *E. coli* which were in contact with the PCPA3 membrane were 96.1 ± 0.4% and 83.7 ± 1.3%, respectively. There were abundant phenolic hydroxyl groups and amine groups in the PCPA coating. The hydroxyl (–OH) in phenol could interact with the cell membrane of the bacteria and disrupt their membrane structures. Amine groups can affect charge distribution in bacterial cells, which make bacterial cells deformed and finally dead [39,40]. As shown in Figure 6c,f, the mortality of the two bacteria in contact with the PCPA3-Ag10 membrane reached 100%, indicating the strong anti-bacterial property of AgNPs.

### 3.5. AgNPs Stability Test

**Figure 7:**
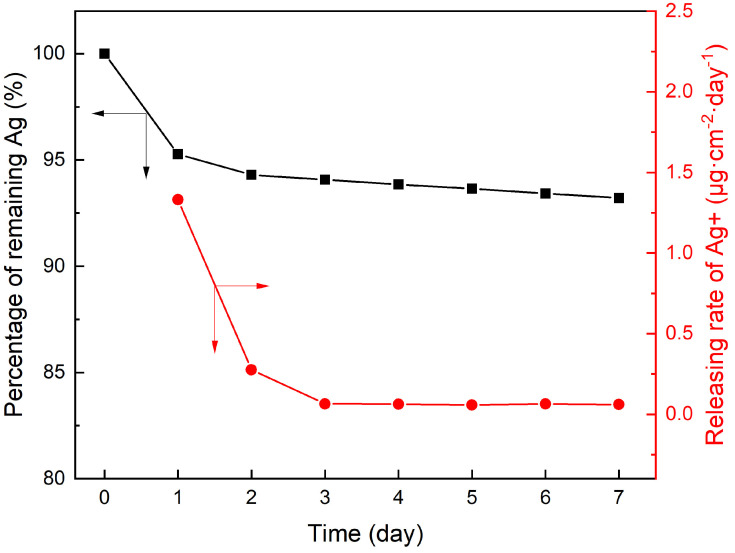
Releasing rates of Ag^+^ from the PCPA3-Ag10 membrane and the weight percentage of AgNPs remaining on the membrane.

Long-term stability was essential for the practical application of the PCPA3-Ag10 membrane. The stability of AgNPs on the membrane was assessed by measuring the amounts of Ag^+^ released in 7 days. As shown in Figure 7, a lot of silver (1.33 μg·cm^−2^·day^−1^) was released into the solution on the first day after the immersion in the NaHCO_3_ solution (pH 8.2). Then, the releasing rate of Ag slowed down to a stable rate (0.06 μg·cm^−2^·day^−1^). The sudden release of silver at the beginning of the immersion may have been due to the loss of AgNPs loosely attached on the membrane surface. It can be seen that there was still about 93.21% silver immobilized on the surface of the membrane after 7 days’ immersion.

We summarize the permselectivity and AgNP stability of the membranes prepared in this work with other similar membranes in Table 5. In general, the anti-bacterial property of all membranes with Ag incorporated in them can be improved. The PCPA3-Ag10 membrane reported here can maintain a long-term and efficient anti-bacterial property with a low release rate of Ag ions. These promising results demonstrate that the strategy of using a PCPA coating as the interlayer to load AgNPs on a membrane surface has a good application perspective.

## 4. Conclusions

In this work, the surface modification of RO membranes was proposed to immobilize AgNPs effectively via the deposition of the poly (catechol/polyamine) interlayer. After the loading of AgNPs, the water flux of PCPA3-Ag10 membrane was reduced to 72.5 ± 0.3 L·m^−2^·h^−1^, which was7.48% less than the virgin membrane. However, in the anti-adhesion experiments with three representative model pollutants, the *FDR_t_* values of the PCPA3-Ag10 membrane were much less than the virgin membrane. Additionally, in the anti-bacterial experiments, the bacterial mortality rates of the PCPA3-Ag10 membrane could reach 100%. The PCPA3-Ag10 membrane showed a strong anti-adhesion property and long-lasting anti-bacterial activity, with a small decrease in the permselectivity. Compared with previous methods of immobilizing AgNPs on RO membranes, the modification method proposed here can improve the anti-adhesion property of membranes and maintain a long-term and stable anti-bacterial ability with a low release rate of Ag ions.

## Figures and Tables

**Table 1:** The EDS elemental compositions.

Sample	Atomic Percent (%)			
	C	N	O	Ag
PCPA3-Ag10	70.03	17.67	10.88	1.42

**Table 2:** The relative surface atomic concentration from XPS.

Samples	Atomic Percent (%)				Atomic Ratio
	C	N	O	Ag	N/O
Virgin	71.10	11.64	17.26	-	0.67
PCPA3	72.37	12.71	14.92	-	0.74
PCPA3-Ag10	69.96	17.06	11.02	1.46	1.35

**Table 3:** The values of *FRR_c_* (c = 1 and 2) and *FDR_t_* of the virgin and PCPA3-Ag10 membranes during the filtration of BSA, SA and DTAB solutions (%).

	BSA		SA		DTAB	
	Virgin	PCPA3-Ag10	Virgin	PCPA3-Ag10	Virgin	PCPA3-Ag10
FRR_1_	92.69 ± 0.11	98.05 ± 0.13	54.43 ± 0.20	98.81 ± 0.42	52.78 ± 2.46	83.23 ± 0.71
FRR_2_	88.68 ± 0.06	95.58 ± 0.02	78.42 ± 0.32	94.53 ± 0.20	49.50 ± 0.75	79.10 ± 0.28
FDR_t_	12.34 ± 0.17	5.63 ± 0.09	36.34 ± 0.28	18.34 ± 0.33	59.81 ± 0.35	34.12 ± 0.15

**Table 4:** The mortalities of bacteria on the membrane samples (%).

Sample	*B. subtilis*	*E. coli*
Virgin	12.5 ± 1.7	15.5 ± 0.9
PCPA3	96.1 ± 0.4	83.7 ± 1.3
PCPA3-Ag10	100	100

**Table 5:** Comparison of performances between Ag-incorporated membranes.

Base Membranes	Modified Layers	Loaded Ag Amounts (μg/cm^2^)	Release Rate of Ag^+^ (μg·cm^−2^·day^−1^)	Water Flux (L·m^−2^·h^−1^)	Rejection (%)	The Mortalities of Bacteria (%)	Refs.
Commercial PSF UF membrane	TA/Fe–Ag TFN	108.30	0.127 ^a^	N: --- M:34.3	N: --- M:98.1	---	[41]
Commercial RO (RE4021-TE, Woongjin Chemical Co.)	TA-Fe-PEI-Ag	52.28	0.080 ^a^	N:45.7 M:52.9	N:98.9 M:99.2	100% for *B. subtilis* and *E. coli*	[27]
Self-made PSU support layer	TFC-S–AgNPs	15.50	0.100 ^b^	N:49.8 M:69.4	N:95.9 M;93.6	---	[21]
Commercial PSF UF membrane	TFC-AgNP@SiO_2_	0.15	0.001 ^b^	N:30.0 M:29.0	N:99.0 M:98.8	92.7, 99.5 and 73.3% for *E. coli*, *P. aeruginosa* and *S. aureus*	[22]
Commercial RO (SW30XL, Dow)	In situ AgNPs	3.70	0.028 ^b^	N:66.5 M: 58.5	N:98.8 M:98.6	78.0%, 91.0%, and 96.0% for *E. coli*, *P. aeruginosa*, and *S. aureus* bacteria colonies	[17]
Commercial RO (ESPA1, Nitto Denko Hydranautics)	PCPA3-Ag10	28.21	0.060 ^a^	N:78.4 M:72.6	N:97.9 M:97.0	100% for *B. subtilis* and *E. coli*	This work

Remark: N, pristine membranes without modification; M, surface-modified membranes; ^a^ the RO membrane samples were immersed in NaHCO_3_ solution; ^b^ the RO membrane samples were immersed in DI water.

## Data Availability

Not applicable.

## Supplementary Materials

The following supporting information can be downloaded at: https://www.mdpi.com/article/10.3390/membranes13050530/s1, Figure S1: The elemental percentage of silver and the silver mass loading were obtained by EDS analysis and silver dissolution experiments; Figure S2: The water fluxes and salt rejection of Virgin, PCPA3 membrane and *PCPA3-Agm* membranes.
