# Supplementary material for: Enhanced Anti-Biofouling Properties of BWRO Membranes via the Deposition of Poly (Catechol/Polyamine) and Ag Nanoparticles
Source: Membranes (Basel). 2023 May 19;13(5):530. doi: 10.3390/membranes13050530 (PMC10223484; doi:10.3390/membranes13050530)
Supplement: Supplementary file 1 [file membranes-13-00530-s001.zip › SI.pdf]

Supporting Information

Enhanced anti-biofouling properties of RO membranes via the deposition of poly (catechol/polyamine) and Ag nanoparticles

Lixin Xie, Yaqian Liu, Shichang Xu* and Wen Zhang*

School of Chemical Engineering and Technology, Tianjin Key Laboratory of Membrane Science and Desalination Technology, State Key Laboratory of Chemical Engineering (Tianjin University), Tianjin University, Tianjin 300350, China
Correspondence: xushichang@sina.com (S. X); zhang_wen@tju.edu.cn (W.Z.)

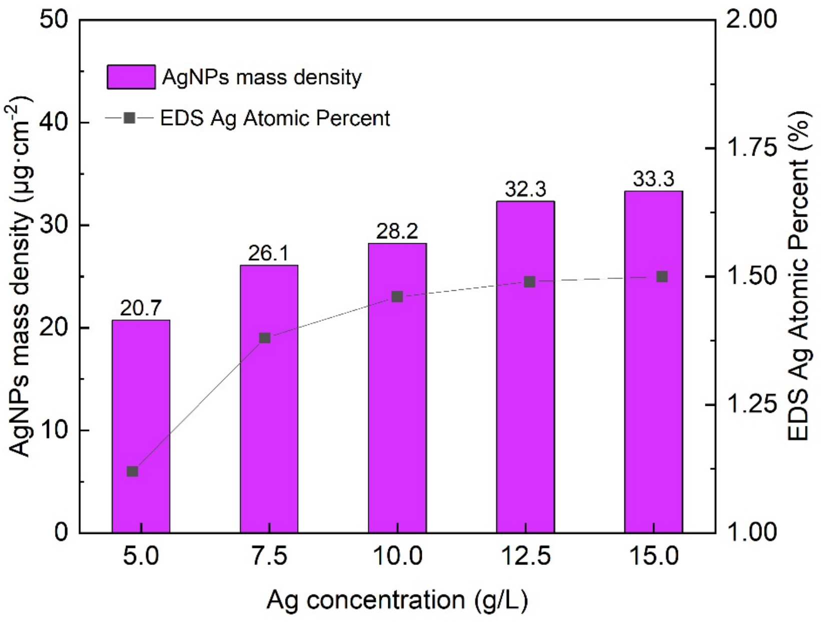

Fig. S1 The elemental percentage of silver and the silver mass loading were obtained by EDS analysis and silver dissolution experiments

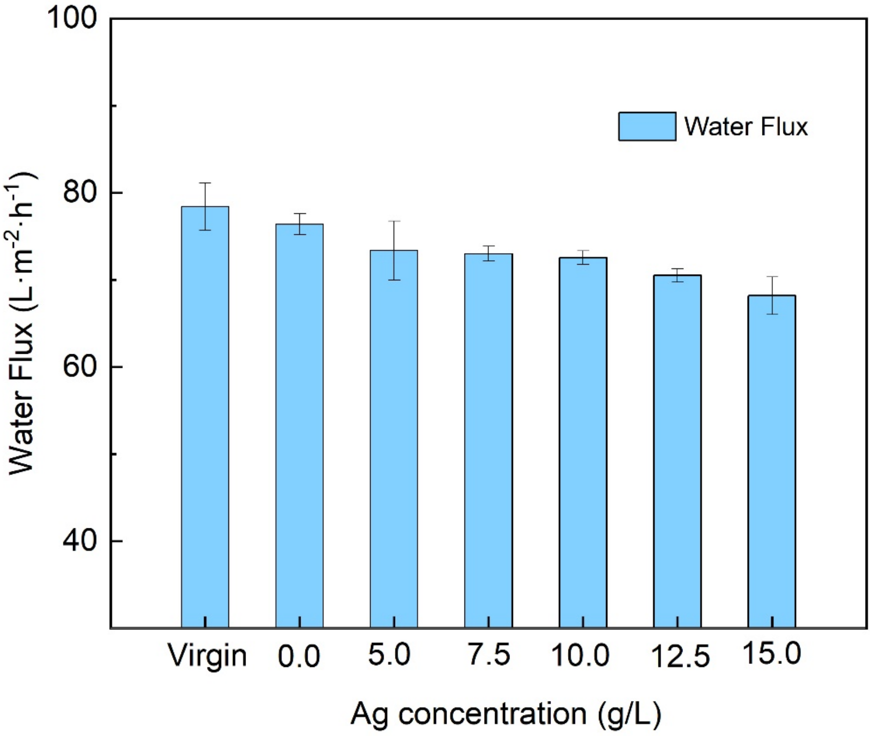

Fig. S2 The water fluxes and salt rejection of Virgin, PCPA3 membrane and PCPA3-Ag*m* membranes
